# PTH immunoassay interference: differential diagnosis with normocalcemic primary hyperparathyroidism?

**DOI:** 10.20945/2359-4292-2023-0315

**Published:** 2024-11-06

**Authors:** Monique Nakayama Ohe, Roberto Massao Takimoto, Claudia M. Aparecida de Francischi Ferrer, Jose Viana Lima, Rosa Paula Biscolla, José Gilberto Henriques Vieira, Maria Izabel Chiamolera

**Affiliations:** 1 Universidade Federal de São Paulo Escola Paulista de Medicina Departamento de Endocrinologia e Metabolismo São Paulo SP Brasil Departamento de Endocrinologia e Metabolismo, Escola Paulista de Medicina, Universidade Federal de São Paulo, São Paulo, SP, Brasil; 2 Grupo Fleury Departamento de Endocrinologia São Paulo SP Brasil Departamento de Endocrinologia, Grupo Fleury, São Paulo, SP, Brasil; 3 Universidade Federal de São Paulo Escola Paulista de Medicina Departamento de Cirurgia de Cabeça e Pescoço São Paulo SP Brasil Departamento de Cirurgia de Cabeça e Pescoço, Escola Paulista de Medicina, Universidade Federal de São Paulo, São Paulo, SP, Brasil

## Abstract

The main diagnostic dilemma in normocalcemic hyperparathyroidism is differentiating this condition from secondary hyperparathyroidism and other causes of elevated parathyroid hormone (PTH) levels in eucalcemic patients, including potential assay interferences. Despite the analytical sensitivity of immunoassays, they may lack adequate accuracy due to several analytical interferences, such as the presence of heterophilic antibodies. Immunoassays for PTH measurement use the immunometric "sandwich" technique, and only a few cases of interference with this assay have been reported to date. We describe herein two patients in whom PTH immunoassay interference was demonstrated. Both patients presented high serum PTH levels, measured using a second-generation Roche electrochemiluminescence assay (ECLIA; Elecsys Roche, Germany), and normocalcemia. When immunoassay interference was suspected, PTH measurements were repeated using a different analytical platform, the 1-84 PTH third-generation Roche Elecsys ECLIA, resulting in normal levels. We subsequently performed serial dilutions using normal mouse serum with the second-generation ECLIA and found no linearity, indicating the presence of interference in both patients. Immunoassay interference may lead to misinterpretation of a patient's results by the laboratory and incorrect treatment planning by the attending physician. Despite its rarity, the presence of interferences in the PTH immunoassay resulting in falsely high PTH levels should be considered when the laboratory result does not match the patient's clinical presentation, thus preventing erroneous diagnoses and unnecessary therapeutic procedures.

## INTRODUCTION

Normocalcemic hyperparathyroidism has become more clearly characterized in recent years (1), and its main diagnostic challenge is differentiating it from secondary hyperparathyroidism. However, other causes of elevated parathyroid hormone (PTH) levels in eucalcemic patients could be responsible for this diagnostic impasse, including assay issues (2-5).

Diagnoses in endocrinology are established on the basis of reliable and accurate biochemical assays along with clinical reasoning. Immunoassays are frequently used to measure both endogenous and exogenous compounds in human serum, forming the basis of many common laboratory tests (4). Immunoassays for PTH measurement use an immunometric "sandwich" technique, with a solid-phase antibody targeting one epitope and a signal antibody targeting a different epitope (3-6). However, sandwich immunoassays are vulnerable to interference (4). These interferences might be due to the presence of heterophilic antibodies, human anti-animal antibodies, autoantibodies, rheumatoid factor, and other proteins (3-7), and can be as high as 6% depending on the type of antibody interference (3,8). A PTH immunoassay interference, which results in falsely high PTH levels, should be considered in the differential diagnosis of normocalcemic hyperparathyroidism and secondary hyperparathyroidism.

We describe herein two patients with demonstrated PTH immunoassay interference, one of whom was referred to surgery (which was not performed) due to a presumptive misguided diagnosis of normocalcemic hyperparathyroidism.

## CASE REPORTS

### Ethical considerations

The report of the present cases was approved by the Fleury Group Research Ethics Committee under the number CAAE: 53303421.3.0000.5474. Both patients signed an informed consent form.

### Patient #1

A 39-year-old white woman was referred to a head and neck surgeon with a presumptive diagnosis of normocalcemic hyperparathyroidism. The patient had no previous symptoms and, during a checkup evaluation, presented high serum PTH levels. She had regular menses and no relevant personal or family history of hyperparathyroidism, kidney stones, or bone disease. Her physical examination was unremarkable. At the time of presentation, she had serum levels of PTH of 373 pg/mL (reference range [RR] 15-65 pg/mL), total calcium of 9.9 mg/dL (RR 8.4-10.2 mg/dL), ionized calcium of 1.29 mmol/L (RR 1.11-1.40 mmol/L), 25-hydroxyvitamin D of 23.9 ng/mL, phosphorus of 4.2 mg/dL (RR 2.5-4.5 mg/dL) and creatinine of 0.94 mg/dL. Biochemical measurements were repeated three times during the following 4 months, and high PTH and normal calcium levels were observed in all measurements (Table 1). Measurement of PTH levels was done using a second-generation Roche electrochemiluminescence assay (ECLIA). The patient also underwent dual-energy X-ray absorptiometry (DXA; GE Lunar DPX NT, GE Healthcare, Madison, WI, USA) examination, which revealed normal values at all sites, including bone mineral density (BMD) and T-score (BMD/T-score) values of, respectively, 1.587 g/cm^2^ and +3.4 in the lumbar spine, 1.334 g/cm^2^ and +2.1 in the femoral neck, and 1.385 g/cm^2^ and +3.0 in the total hip. A diagnosis of normocalcemic hyperparathyroidism was suspected after ruling out the most common causes of secondary increases in PTH levels, such as vitamin D deficiency or insufficiency, very low calcium intake, idiopathic hypercalciuria, impaired renal function, malabsorption, and use of medications. A second opinion was sought from an endocrinologist specializing in bone metabolism disease. Upon review of the presence of normal mineral bone density by DXA examination in all sites, the absence of hypercalciuria, and the lack of history of kidney stones or their presence on ultrasound examination, the diagnosis of normocalcemic hyperparathyroidism seemed unlikely. At this stage, the possibility of PTH immunoassay interference was considered.

**Table 1:** Biochemical measurements over time in Patient #1

	11/2020	01/2021	02/2021	03/2021
**PTH** (pg/mL)	373	221	427	318
**tCa** (mg/dL) & **iCa** (mmol/L)	9.9 & N/A	N/A	9.8 & 1.25	9.1 & 1.29
**25-OHD** (ng/mL)	23.9	44.7	51	N/A
**P** (mg/dL)	4.2	N/A	4.3	3.7
**ALP** (U/L)	N/A	N/A	47	N/A
**Cr** (mg/dL)	N/A	N/A	N/A	0.82
**24-h UCa** (mg/24 h)	N/A	N/A	N/A	135

Abbreviations: 24-h UCa, 24-hour urinary calcium; 25-OHD, 25-hydroxyvitamin D; ALP, alkaline phosphatase; Cr, serum creatinine; P, phosphorus; PTH, parathyroid hormone; tCa & iCa, total calcium and ionized calcium. Reference values: PTH, 10-65 pg/mL; tCa, 8.6-10.3 mg/dL; iCa, 1.11-1.40 mmol/L; P, 2.5-4.5 mg/dL; AP, 35-104 U/L; Cr, 0.6-1.10 mg/dL; Uca, 55-220 mg/24 h.

### Patient #2

A 46-year-old white woman with a medical history of recurrent kidney stones presented with serum levels of PTH of 2,906 pg/mL (RR 15-65 pg/mL), total calcium of 9.5 mg/dL (RR 8.4-10.2 mg/dL), ionized calcium of 1.30 mmol/L (RR 1.11-1.40 mmol/L), 25-hydroxyvitamin D of 16 ng/mL, phosphorus of 3.5 mg/dL (RR 2.5-4.5 mg/dL) and creatinine of 0.73 mg/dL. She had no other relevant complaints in her medical history, including bone disease or fractures. Biochemical measurements were repeated and confirmed. Given the extremely high PTH levels, the possibility of assay interference was considered.

### Repeat analyses in Patients #1 and #2

In both patients, serum PTH levels were initially measured using a second-generation ECLIA (Elecsys, Roche, Germany). When the possibility of interference in the PTH immunoassay was suspected, the analysis was repeated using the 1-84 PTH third-generation Roche Elecsys ECLIA. With the third-generation equipment, the PTH concentrations were within the normal range: 16.1 pg/mL in Patient #1 and 36 pg/mL in Patient #2 (RR 20-58 pg/mL). In subsequent analysis using the second-generation ECLIA equipment and serial dilutions using normal mouse serum, the measurements showed no linearity, confirming the presence of interference (Table 2).

**Table 2:** Parathyroid hormone measurements after dilution using normal mouse serum

Patient	PTH levels (in pg/mL)			
	Nodilution	1:2dilution	1:5dilution	1:10dilution
**#1**	413.5	14.06	16.65	N/A
**#2**	2906	N/A	15	<3

Abbreviation: PTH, parathyroid hormone. The PTH levels were measured using an electrochemiluminescence assay (ECLIA; Elecsys Roche, Germany), with reference values of 15-65 pg/mL.

## DISCUSSION

Primary hyperparathyroidism (PHP) is a common endocrine disorder associated with hypercalcemia and caused by excessive PTH secretion. Over time, the clinical presentation of primary hyperparathyroidism has evolved from being predominantly symptomatic to increasingly less symptomatic or even asymptomatic, including in developing countries (9). In the early 2000s, the variability of the clinical presentation of primary hyperparathyroidism broadened further with reports of patients who appeared to have primary hyperparathyroidism due to persistently elevated PTH concentrations but serum calcium concentrations persistently within the normal range, referred to as normocalcemic primary hyperparathyroidism (10,11).

**Figure 1:**
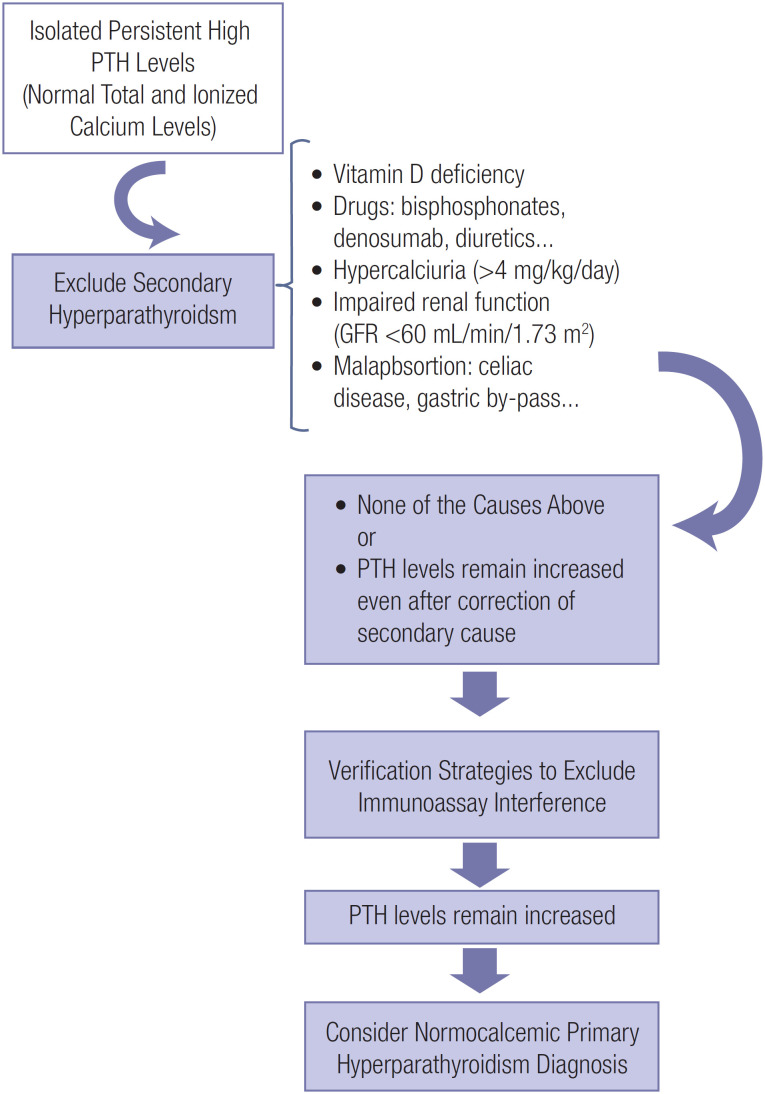
Differential diagnosis of high isolated parathyroid hormone (PTH) levels. Abbreviations: GFR, glomerular filtration rate; PTH, parathyroid hormone.

Secondary causes of increased PTH concentrations (*e.g.*, vitamin D deficiency, malabsorption syndromes, renal insufficiency, hypercalciuria, and use of lithium and thiazide diuretics) should always be investigated (11), along with the use of medications for osteoporosis treatment, such as bisphosphonates and denosumab (12). After excluding these secondary causes of elevated PTH levels, the diagnosis of normocalcemic primary hyperparathyroidism can be established (11) (Figure 1).

Patient #1 was referred for surgery due to a presumptive diagnosis of normocalcemic hyperparathyroidism. However, despite persistently high serum PTH levels (up to 400 pg/mL), the possibility of immunoassay interference was considered after verifying normal bone mass measurements on DXA and ruling out secondary causes of isolated PTH elevation, symptoms, kidney stones, and bone disease. In Patient #2, the hypothesis of assay interference was raised due to the extremely high PTH levels without correlation with other laboratory tests or clinical presentation.

Immunoassays are commonly used for diagnosing and following up several diseases in endocrinology. Despite substantial advances in their reliability, analytical interference remains a major and often underestimated problem (13). Immunoassays are inherently vulnerable to immunoglobulin-related interferences (14). The prevalence of PTH interferences ranges from 0.5% to 6%, depending on the assay (15). Only a few reports in the literature have described asymptomatic elevated PTH levels as a result of immunoassay interference (3,4,16,17), and heterophilic antibodies are the most common cause of such interference in two-site immunoassays (8). Heterophilic antibodies are defined as endogenous antibodies that bind external antigens (13). These antibodies may occur naturally without an identifiable cause or may result from infection, vaccinations, contact with animals, or the use of animal immunoglobulins therapeutically or for in vivo diagnosis (13,18). Interestingly, both patients were business professionals and none of them were exposed to rodents or other animals. Classically, heterophilic antibodies are polyspecific and bind with weak affinity to numerous antigens. Autoantibodies, such as rheumatoid factor, may also act as interfering heterophilic antibodies (13). Cavalier and cols. (2), in a series of 734 patients with elevated PTH levels, reported that 3.4% presented interference due to heterophilic antibodies and 1.2% presented interference due to rheumatoid factor (2,3). Additionally, human anti-animal antibodies have been described as binding with high specificity and affinity to animal antigens, such as mouse immunoglobulins (14). The prevalence of potentially interfering heterophilic antibodies in the general population is estimated to be about 30%-40% based on serum samples (19,20), although analytically important interferences are reported only in 0.5%-3% (21) and as high as 6% (15). Interfering substances may also impact other components of the assay; for example, biotin supplementation is a frequent cause of interference in streptavidin-biotin-based immunoassays (22).

**Figure 2:**
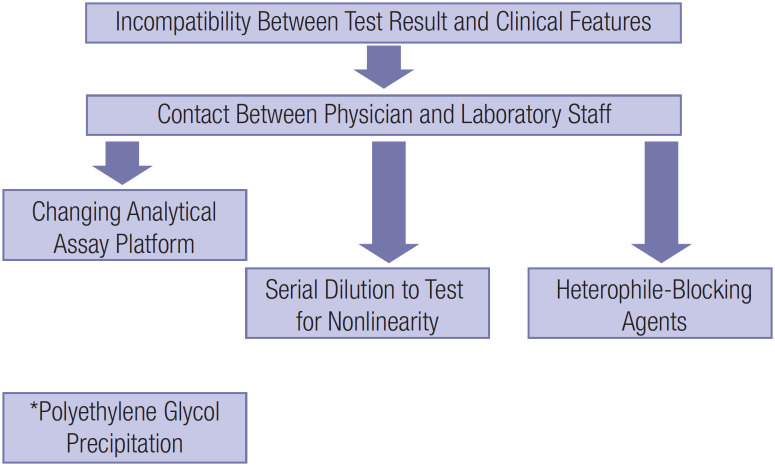
Recommended flowchart for investigating suspected falsely elevated parathyroid hormone (PTH) levels due to heterophile antibodies. The first step is to rerun the assay with a different immunoassay, followed by serial dilutions to evaluate linearity and/or pretreatment with heterophile-blocking agents or tubes. *Polyethylene glycol precipitation could also be used.

Assay interference is usually suspected in the presence of discrepancies between laboratory results and clinical features. While multiple strategies are available to confirm or exclude the occurrence of heterophilic antibodies (13), no universal strategy is available to detect assay interference (3). In Figure 2, the present authors propose a practical approach for investigating the possibility of falsely elevated PTH levels. One strategy is to measure PTH levels on different analytical platforms, while the other is to treat the sample with heterophile-blocking agents or precipitate the sample with polyethylene glycol (PEG). Another approach is to perform serial dilution to test for linearity (14). This may be accomplished with diluents provided by the manufacturer, although no specific diluent is available for Roche's PTH assay. The strategy to use mouse serum is more related to its heterophile-blocking action since commonly used heterophile-blocking tubes are unavailable in our country.

In this study, high serum PTH levels were observed using the intact PTH (second-generation) ECLIA from Roche. We repeated PTH measurement using a different analytical platform from the same manufacturer, the 1-84 PTH (third-generation) ECLIA from Roche, and the PTH concentrations obtained were within the normal range. Although the platform is the same, the configuration of the antibodies is different between the two assays. Third-generation immunometric assays (*e.g*., the bio-intact PTH assay) use an amino-terminal-specific antibody directed to the first four PTH amino acids. This approach ensures that only the active molecular form (*i.e*., 1-84) is measured, excluding recognition of long carboxyl-terminal fragments detected by previous PTH assays. We also performed serial dilutions with normal mouse serum using the second-generation ECLIA used first in both patients, and the results showed no linearity, indicating the presence of interference.

This study has some limitations, including the fact that none of the patients were tested for the presence of rheumatoid factor or monoclonal gammopathy, both of which can cause similar immunoassay interference. However, the patients had no complaints or signs of diseases related to these biomarkers. Additionally, we did not test other proper diluents because there are no specific agents for Roche's PTH test. We also did not test analytical platforms from other manufacturers or perform PEG precipitation for further confirming our findings.

Immunoassay interference may lead to a misinterpretation of the patient's results by the laboratory and incorrect treatment planning by the attending physician. Laboratory values that are inconsistent with the patient's clinical presentation are key findings, and their identification depends on close communication between physicians and laboratory staff (3). Despite its rarity, interferences in PTH immunoassays resulting in falsely high PTH levels should be considered when the laboratory result does not match the patient's clinical presentation, preventing erroneous diagnosis and unnecessary therapeutic procedures.

In conclusion, the occurrence of PTH immunoassay interference, resulting in falsely high PTH levels, should be considered during the investigation of secondary and normocalcemic primary hyperparathyroidism, particularly in patients with no other abnormal laboratory results that could justify the elevated PTH levels.
